# Metal Complexes in Diagnosis and Therapy

**DOI:** 10.3390/ijms23084377

**Published:** 2022-04-15

**Authors:** Diego Tesauro

**Affiliations:** Interuniversity Research Centre on Bioactive Peptides (CIRPeB), Department of Pharmacy, University of Naples “Federico II”, via Montesano, 49, 80131 Naples, Italy; diego.tesauro@unina.it; Tel.: +39-0812536643

The use of metal complexes for health and healing has been in use for over the last several millennia and perhaps longer. Transition metals have a long history as antibacterials and antivirals. Gold-based drugs were being used in China and the Middle East as far back as 3500 years ago; drinking water was disinfected by storing it in silver pots in the Persian empire, and mercurous chloride (Hg_2_Cl_2_) was an active diuretic during the Renaissance period. In the previous century, fields of applications expanded, and in this one, many therapeutic treatments for most widespread diseases have been based on inorganic drugs [1]. 

Metallodrugs can be a metal complex that is itself the active agent and a carrier of active principles. Moreover, in metal medicine, drugs themselves do not contain a metal but act on native biometals or metal-containing biomolecules or influence metal-trafficking pathways in ways that affect cellular processes. Exploiting the power of all of these classes for therapeutic or diagnostic use requires the understanding of properties of metal complexation in a biological context. The ability to form complexes with different coordination numbers, geometries, and varied oxidation number and the ability to bind to biomolecules are some features that prove the potential of metal ions as exciting means to explore innovation in drug discovery. The success of simple inorganic compounds pushed in the last 50 years is based on structure and properties related to the design of new metal complexes to influence the properties, speciation, reactivity, and ultimately biological effect in the given medicinal context. In different biological and coordination environments, the same metal ion can have very different coordination pathways influencing biological processes interacting with biological molecules. Advances in bioanalytical techniques improved the knowledge of metallomics and have opened unprecedented opportunities. In recent decades, insights into metal ion homeostasis have grown exponentially, and a more systemic understanding of metal ion intoxication and homeostasis has been acquired [2,3]. Therefore, now scientists have much more detail about the molecular behavior of many enzymes, transporters, and metallochaperones involved, including numerous biomolecular interactions and their regulation. This knowledge, in turn, provides a spotlight to bring new therapeutic prospects into focus. In this issue, Santoro et al. [4] increased the knowledge about the binding mode of copper and zinc ions with TetraHPRG, a 20-aa-containing peptide belonging to the H/P domain, on the binding of kininogen (HKa) with tropomyosin. The chemical physical characterizations based on potentiometric study and circular dichroism show the formation of complex species involving imidazole amide nitrogen atoms in metal binding. These results indicate that both metal ions are crucial in interacting with TetraHPRG, tropomyosin, and HKa. The clinical achievement of inorganic drugs, such as cisplatin and its derivate chemotherapeutics agents, has significantly improved the study of metal ions on the biological system to treat several diseases [5].

Radiation-based anticancer therapy, such as proton therapy (PT), can be employed to reduce tumors before surgical intervention [6]. The application of bimetallic NPs can support the effect of proton irradiation acting as a radiosensitizer, increasing effects. Klebowski et al. [7] synthesized novel, fancy-shaped bimetallic gold–platinum nanocauliflowers (AuPt NCs) with a highly developed surface area. They examined the radio-sensitizing effect of NCs on three cancer cell lines with different malignancy: HCT116, SW480, and SW620, as well as the normal colon cell line FHC. Authors detected a significant reduction in cancer cell viability compared to normal cells. Thus, the radio-enhancing features of AuPt NCs indicate their potential application for the improvement in effectiveness of anticancer proton therapy. The metal can increase the chemotherapeutic properties of organic molecules such thiosemicarbazones [8]. Pitucha et al. [9] prepared a series of thiosemicarbazone derivatives and their Cu (II) complexes. The antitumor activity of fully characterized complexes with physical chemical methods was tested in vitro on G361, A375, and SK-MEL-28 human melanoma cells and BJ human normal fibroblast cells. The cytotoxicity was assessed with cell cycle analysis and apoptosis/necrosis detection, establishing an association with DNA damage and the G2/M phase of cell cycle arrest and disorders of the expression of antioxidant enzymes. Metal centers play a role as a contrast agent for early diagnosis based on contrast agents able to discriminate pathological tissues. Paramagnetic gadolinium(III) ion allows for increasing the relaxivity of water protons, improving the resolution in magnetic resonance imaging (MRI) [10,11]. Orts-Arroyo et al. [12] synthesized new gadolinium-based contrast agents centered on thymine nucleobase of formula [Gd(thy)_2_(H_2_O)_6_](ClO_4_)_3_·2H_2_O (1) [thy = 5-methyl-1H-pyrimidine-2,4-dione or thymine, exhibiting larger relaxivity values than current clinically employed complexes. Its magnetic and relaxometric properties have been investigated using SQUID magnetometer and MRI phantom studies. Enhanced properties are due to the higher number of coordinate water molecules than the typical commercial contrast agents. Moreover, specific targeting might provide higher resolution and better functional images. 

Metals can be carried out as theranostics, a combination of the terms therapeutics and diagnostics. Magnetic nanoparticles (MNPs) can tune the magnetic field in their environment, which positively impacts theranostic applications in nanomedicine [13]. In recent years, the application of magnetic nanomedicine as theranostic devices has garnered enormous attention in cancer treatment research. Mkhatshwa et al. [14] reviewed novel cancer theranostics focusing on potential applications of nanoparticles (i.e., magnetite Fe_3_O_4_) functionalized with the Re(I) tricarbonyl complexes as a bimodal contrast agent for MRI and optical imaging of nanoparticles. This combination of Re(I) with MNPs can improve low distribution and cell penetration into deeper tissues.

The regained and growing interest in metal complexes’ antimicrobial effect is related to antibiotic resistance, which represents a health and social problem worldwide, and is a significant hazard for humankind in the near future, as recently recognized by the World Health Organization [15]. Metals have been able to act as antibacterials since the early eras. Early annals report water stored in silver pots to prevent bacterial growth [16]. The success of this metal lies in its effectiveness at low concentrations, in its low toxicity, and lastly, in the lack of resistance. The active specie is the Ag(I) ion that coordinates biomolecules. The stability of the complex must be able to release the ion, and NHC-carbene ligands own the best properties to perform this task [17]. Prencipe et al. [18] prepared two NHC-carbenes based on acridine scaffold, antimicrobial ligand, and detailed nonclassical-pyrazole-derived mono NHC-Ag neutral and bis NHC-Ag cationic complexes. Imidazolium NHC silver complex containing the acridine chromophore showed effectiveness at extremely low MIC values (≤1 μM) against two Gram-negative (*E. coli* and *P. aeruginosa*) and two Gram-positive bacteria (*B. subtilis* and *S. aureus*). Although pyrazole NHC silver complexes are less active than the acridine NHC-silver, they represent the first example of this class of compounds with antimicrobial properties. 

Moreover, all complexes at 4 h are not toxic and they show very low activity against mammalian cells (Hek lines) after 24 h. Antimicrobial properties can be performed by metal ions which play essential roles. Cobalt is an essential trace element present in the human body, but a small number of Co(III) complexes are known as inorganic pharmaceuticals. Only one containing a Schiff base named CTC-96 (Doxovir) [19] has been tested in a phase II clinical trial for antiviral treatment against herpes simplex virus (HSV) [20]. Laísa de P. Fernandes et al. [21] expected to provide new structural information and biological insights of Co(III) thiosemicarbazone-based compounds. They synthesized two known Schiff base (2-acetyl-piridine-*N*(4)-R-thiosemicarbazone (R = methyl and phenyl) Co(III) complexes to design new structural experimental studies together with the antibacterial and antiviral activity. The screening complexes in seven bacteria strains and on the chikungunya virus revealed a remarkably high potency for the phenyl group and revealed promising MIC and MBC values which ranged from 0.39 to 0.78 µg/mL compared to the methyl-derived group. Moreover, further investigations of the interactions between the complex and bacteria through molecular docking analysis support future studies on this molecule and the good potentiality of Co(III) complexes as an inorganic drug. Bismuth-based compounds are in clinical use to treat gastrointestinal diseases and their gastroprotective effects. Moreover, they effectively treat *H. pylori* infection and also have broad antimicrobial, antileishmanial, and anticancer properties. Characteristics of Bi(III) ion are the coordinative flexibility and the wide variety of coordination structures affected by the different coordination modes as carboxylate ligands [22]. Exploiting these properties, Kowalik et al. [23] prepared two novel coordination polymers, [Bi_2_(2,3pydc)_2_(2,3pydcH)_2_(H_2_O)]_n_ and {(Et_3_NH)_2_[Bi(2,3pydc)(2,3pydcH)Cl_2_]}_n_, using as a prolinker pyridine-2,3-dicarboxylic acid (2,3pydcH_2_). The first polymer is more active against *H. pylori* than the other. Moreover, it was detected that the polymer was more than twice as active as the second polymer. It can be concluded that there is an existing relationship between the structure and the antibacterial activity because the presence of chloride and triethylammonium ions in the structure of the complex reduces the antibacterial activity. 

The beneficial properties of zinc compounds for the skin and their role as an active substance in dermatological products are well known [24]. Abendrot et al. [25] developed new antiacne preparations containing zinc glycine and histidine complexes as active ingredients. These formulations (ZnGly and ZnHis gel formulations) were physicochemically stable and demonstrated high microbiological purity with a well-selected preservative system. Moreover, no irritation or allergy was observed. These preparations containing Zn(II) complexes can be topically used in the treatment of acne skin due to their high antibacterial activity against *C. acnes* and low cytotoxicity for the skin cells. Another application of inorganic drugs may be used against inflammatory diseases. Krajewsk et al. [26] proposed a new concept for anti-inflammatory bowel diseases (IBD) and proved that Au(III) complexes may have therapeutic potential in the treatment of IBD. This is the first study proving that Au(III) complexes may have therapeutic potential in IBD treatment. The new Au(III) complex TGS 121 was designed and was screened using in in vitro studies using a mouse macrophage cell line, RAW264.7, and in vivo, in the dextran sulphate sodium (DSS)-induced mouse model of colitis. The mechanism of action of TGS 121 was related to the enzymatic and non-enzymatic antioxidant system; in fact, TGS 121 induced changes in the tight junction complexes’ expression in the intestinal wall.

In conclusion, this Special Issue addressing inorganic drugs focused on wide aspects, from the design, characterization, evaluation, and development of novel metal complexes able to interact with biological tissues in order to fight diseases, to cancer, infections, and inflammations. 
